# Knockdown of UBQLN1 Functions as a Strategy to Inhibit CRC Progression through the ERK-c-Myc Pathway

**DOI:** 10.3390/cancers15123088

**Published:** 2023-06-07

**Authors:** Ruoxuan Ni, Jianwei Jiang, Mei Zhao, Shengkai Huang, Changzhi Huang

**Affiliations:** 1 Department of Etiology and Carcinogenesis, National Cancer Center/National Clinical Research Center for Cancer/Cancer Hospital, Chinese Academy of Medical Sciences and Peking Union Medical College, Beijing 100021, China; 18253163755@163.com (R.N.);; 2 State Key Laboratory of Molecular Oncology, National Cancer Center/National Clinical Research Center for Cancer/Cancer Hospital, Chinese Academy of Medical Sciences and Peking Union Medical College, Beijing 100021, China; 3 Beijing Key Laboratory for Carcinogenesis and Cancer Prevention, National Cancer Center/National Clinical Research Center for Cancer/Cancer Hospital, Chinese Academy of Medical Sciences and Peking Union Medical College, Beijing 100021, China; 4 The First Affiliated Hospital, Institute of Translational Medicine, Zhejiang University School of Medicine, Hangzhou 310058, China; 5 Department of Clinical Laboratory, National Cancer Center/National Clinical Research Center for Cancer/Cancer Hospital, Chinese Academy of Medical Sciences and Peking Union Medical College, Beijing 100021, China

**Keywords:** UBQLN1, ERK-c-Myc pathway, colorectal cancer, cancer progression

## Abstract

**Simple Summary:**

The mortality rate of CRC is higher than that of other malignant tumors because of its high late-diagnosis rate. Searching for new diagnostic biomarkers for CRC is very important clinically. Accumulating evidence has demonstrated that UBQLN1 plays an important role in many biological processes. However, the role of UBQLN1 in CRC progression is still elusive. In this study, we found that UBQLN1 was significantly highly expressed in CRC tissues compared with normal tissues. In addition, reduced UBQLN1 inhibited CRC cell proliferation, colony formation, and EMT in vitro and CRC cells’ tumorigenesis and metastasis of nude mice in vivo. Moreover, the knockdown of UBQLN1 reduced the expression of c-Myc by downregulating the ERK-MAPK pathway. Collectively, the knockdown of UBQLN1 inhibits the progression of CRC through the ERK-c-Myc pathway, which provides new insights into the mechanism of CRC progression. UBQLN1 may be a potential prognostic biomarker and therapeutic target of CRC.

**Abstract:**

Purpose: Colorectal cancer (CRC) is characterized by the absence of obvious symptoms in the early stage. Due to the high rate of late diagnosis of CRC patients, the mortality rate of CRC is higher than that of other malignant tumors. Accumulating evidence has demonstrated that UBQLN1 plays an important role in many biological processes. However, the role of UBQLN1 in CRC progression is still elusive. Methods and results: we found that UBQLN1 was significantly highly expressed in CRC tissues compared with normal tissues. Enhanced/reduced UBQLN1 promoted/inhibited CRC cell proliferation, colony formation, epithelial–mesenchymal transition (EMT) in vitro, and knockdown of UBQLN1 inhibited CRC cells’ tumorigenesis and metastasis in nude mice in vivo. Moreover, the knockdown of UBQLN1 reduced the expression of c-Myc by downregulating the ERK-MAPK pathway. Furthermore, the elevation of c-Myc in UBQLN1-deficient cells rescued proliferation caused by UBQLN1 silencing. Conclusions: Knockdown of UBQLN1 inhibits the progression of CRC through the ERK-c-Myc pathway, which provides new insights into the mechanism of CRC progression. UBQLN1 may be a potential prognostic biomarker and therapeutic target of CRC.

## 1. Introduction

Colorectal cancer (CRC) is a globally important disease that ranks as the third most diagnosed malignancy worldwide [1,2,3]; it has the second highest incidence among malignant tumors in China and ranks first among digestive tract tumors, according to the latest report from the National Cancer Center [4]. For a long time, due to the frequent diagnosis of CRC at an advanced stage, mortality ranks second among cancers globally [5,6]. Therefore, it is critical to find novel biomarkers for early diagnosis, as well as new directions for the treatment of CRC.

UBQLN1 belongs to the family of ubiquitin-like proteins and plays an important role in regulating protein degradation [7,8]. In eukaryotes, UBQLN1 connects proteasomes and ubiquitinated proteins to stimulate the degradation of ubiquitinated and misfolded proteins via autophagy regulation [9,10]. The inactivation of UBQLN1 function can induce the pathological process of a variety of human neurodegenerative diseases, such as Alzheimer’s disease and Huntington’s disease [11,12]. In addition, UBQLN1 is related to the occurrence and progression of a variety of human tumors. UBQLN1 is abnormally upregulated in breast cancer, and the knockdown of UBQLN1 inhibited the invasion and stemness of breast cancer cells through the AKT pathway [13]. In non-small cell lung carcinoma, loss of UBQLN1 repressed EMT [14]. Upregulated UBQLN1 predicts a poor prognosis in hepatocellular carcinoma patients and induced PGC1β degradation in a ubiquitination-independent manner to reduce mitochondrial biogenesis [15]. However, the expression of UBQLN1 in colorectal cancer and the corresponding mechanism of action have not yet been reported.

KRAS mutation is found in approximately 35–45% of colorectal cancers [16]. Mutant KRAS was reported to cause activation of the ERK signaling pathway [17]. Previous research has shown that activation of the ERK pathway could promote c-Myc protein stability by post-translational phosphorylation [18].

In this study, we noted enhanced expression of UBQLN1 in CRC samples compared with normal samples. Moreover, we found that overexpression/knockdown of UBQLN1 promoted/inhibited the proliferation, migration, invasion, and epithelial–mesenchymal transition (EMT) of CRC cells in vitro and knockdown of UBQLN1 inhibited CRC cell’s tumorigenesis as well as metastasis in vivo. Furthermore, we demonstrated that the knockdown of UBQLN1 inhibited cell progression by downregulating the ERK-c-Myc signaling pathway.

## 2. Materials and Methods

### 2.1. Cell Lines and Cell Culture

HCT-116, SW480, and HEK-293T cells were preserved by Huang Changzhi’s laboratory and cultured in DMEM with 10% fetal bovine serum (FBS). DLD1, HCT-8, LoVo cell was preserved by Huang Changzhi’s laboratory and cultured in RPMI-1640 with 10% FBS. All cells were grown at 37 °C with 5% CO_2_ in cell incubator.

### 2.2. Plasmid Constructions

Human cDNA of Ubqln1 was cloned using Q5 high-fidelity DNA polymerase Kit (New England Biolabs, MA, USA). The full-length cDNA of Ubqln1 was constructed into pLVX-Puro vector. The Ubqln1 full-length primers were as follows: forward, 5′-AAGTCTAGAGAATTCGGATCCATGGCCGAGAGTGGTGAAAGC-3′; reverse, 5′-AACAAGCTTCCATGGCTCGAGCTATGATGGCTGGGAGCCCAG-3′. Short hair-pin RNA (shRNA) targeting Ubqln1 was initially inserted into the BamH I and EcoR I sites of pSIH1 plasmid, forming the pSIH1-shUBQLN1 plasmids. Three pairs of shUBQLN1 sequences were as follows: shUBQLN1.1: sense: 5′-GTTTTTCAATGTCTAAGTCGTCCCAAAAGAGAACTTTTTGGGACGACTTAGACATTGCCTAG-3′; antisense: 5′-GCAATGTCTAAGTCGTCCCAAAAAGTTCTCTTTTGGGACGACTTAGACATTGAAAAACTTAA-3′; shUBQLN1.2: sense: 5′-GTTTTTGAGGGTTGAAAGGAGGTTGTTAGAGAACTTAACAACCTCCTTTCAACCCTCCCTAG-3′; antisense: 5′-GGAGGGTTGAAAGGAGGTTGTTAAGTTCTCTAACAACCTCCTTTCAACCCTCAAAAACTTAA-3′; and shUBQLN1.3: sense: 5′-GTTTTTGGAGTCGATGTCTTAGGTCTTAGAGAACTTAAGACCTAAGACATCGACTCCCCTAG-3′; antisense: 5′-GGGAGTCGATGTCTTAGGTCTTAAGTTCTCTAAGACCTAAGACATCGACTCCAAAAACTTAA-3′.

Human cDNA of c-Myc was cloned using Q5 high-fidelity DNA polymerase Kit (New England Biolabs, MA, USA). The full-length cDNA of c-Myc was constructed into pLVX-Puro vector. The c-Myc full-length primers were as follows: forward, 5′-CCGGAATTCCTGGATTTTTTTCGGGTAGTG-3′; reverse, 5′-CCGCTCGAGTTACGCACAAGAGTTCCGTAG-3′.

### 2.3. Establishment of Stable Expression Cell Lines

Lentivirus was produced using packaging system psPAX2, pMG2G, and pLVX-Ubqln1-Puro (pSIH1-shUBQLN1) plasmid at the ratio of 4:2:4, transfected by Lipofectamine 2000 (Invitrogen) in HEK-293T cells.

Cells were plated in 6-well plates and infected with lentivirus assisted by 8 μg/mL poly-brene (Sigma-Aldrich, St. Louis, Missouri, USA) for 36 h, and were then selected by puromycin for two weeks. Expression of Ubqln1 in stable cell lines was verified by Western blot. 

### 2.4. Cell Counting Kit-8 (CCK-8) Assay

CCK-8 assay was carried out to assess cell proliferation. CCK-8 reagent (Meilunbio, Dalian, China) was added to 96-well plates with cells seeded in at a ratio of 1:10, and then spectrometric absorbance at 450 nm was measured after incubation at 37 °C for 1 h.

### 2.5. Colony Formation Assay

For colony formation assay, every 400 cells were seeded into 6-well plate and then incubated at 37 °C until colonies were macroscopic. Next, colonies were stained with 0.5% crystal violet, and the number of colonies was counted.

### 2.6. Cell Invasion and Motility Assay

To investigate cell motility and invasion capabilities, a total of 2 × 10^5^ colorectal cancer cells were added to the upper chamber of cell culture insert (pore size, 8 μm; Corning, NJ, USA) coated with diluted Matrigel basement membrane matrix (BD, Franklin Lakes, NJ, USA) and grown in serum-free medium. In the lower chamber, 600 μL of cell culture medium supplemented with 10% FBS was added. As a result of PBS wash, non-attached cells were removed after being incubated for 24 h in 5% CO_2_ at 37 °C. We fixed attached cells in 4% paraformaldehyde for 30 min and stained them with 0.5% crystal violet. Five visual fields at ×100 magnification were randomly selected, and the number of cells in fields was recorded. The mean value was calculated from three independent experiments performed in triplicate.

### 2.7. Western Blot

Cells were washed with ice-cold PBS and lysed in lysis buffer. BCA protein concentration determination was used to quantify total protein contents. A total of 20 μg of protein were loaded on SDS-PAGE and transferred to PVDF membrane. After blocking in 5% BSA, PVDF membrane was incubated with specific primary antibodies (anti-β-actin, 1:1000, Abclonal Technology, Wuhan, China; anti-UBQLN1, 1:1000, Proteintech, Wuhan, China; anti-GAPDH, anti-E-cadherin, anti-MMP-9, anti-VIMENTIN, anti-t-ERK1/2, anti-p-ERK1/2, anti-MEK1, anti-p-MEK1, and anti-c-MYC, 1:1000, Cell Signaling Technology, MA, USA) overnight. After washing, membrane was incubated with secondary antibody for 1 h. Finally, blots were visualized with enhanced chemiluminescent (NCM Biotech, Suzhou, China) by GE ImageQuant LAS 4000.

### 2.8. Nude Mice Xenograft and Metastasis Experiments

Nude mice (5 weeks old) were purchased from Beijing Huafukang Bioscience and raised in SPF laboratory animal room. All animal experiments were approved by the Animal Care and Use Committee of the Chinese Academy of Medical Sciences Cancer Hospital, and conducted in accordance with guidelines of the National Animal Welfare Law of China.

In nude mice xenograft experiment, LoVo-shCTRL and LoVo-shUBQLN1.2 cells at their exponential growth phase were harvested and washed twice in 0.9% saline water, and then resuspended in 0.9% saline water at a density of 3 × 10^7^ cells/mL. Cell suspension (0.1 mL, 3 × 10^6^ cells) was subcutaneously injected into the right flank of 5- to 6-week-old male BALB/c nude mice (4 mice in each group). Mice were humanely euthanized when the subcutaneous tumors reached 10 mm in diameter.

In nude mice metastasis experiment, LoVo-shCTRL and LoVo-shUBQLN1.2 cells at their exponential growth phase were harvested and washed twice in 0.9% saline water, and then resuspended in 0.9% saline water at a density of 1.5 × 10^7^ cells/mL. Cell suspension (0.1 mL, 1.5 × 10^6^ cells) was injected into tail veins of nude mice (5 mice in each group). Mice were humanely euthanized when the mice were raised to 40–50 days.

### 2.9. Statistical Analysis

Data were described as mean ± SD from at least 3 independent experiments. Student’s *t*-test was used to assess statistical differences between groups. Differences with *p* value less than 0.05 were considered to be statistically significant. Statistical analysis of data was performed using GraphPad Prism 8.0 and SPSS 17.0 software.

## 3. Results

### 3.1. UBQLN1 Enhanced Expression in Colorectal Cancer Tissues and Is Correlated with Poor Prognosis

**Figure 1:**
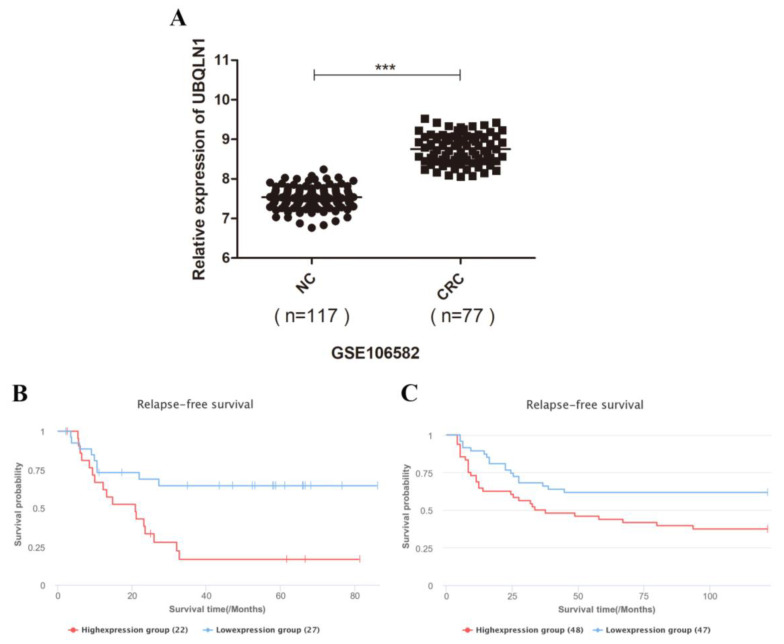
UBQLN1 enhanced expression in colorectal cancer tissues and is correlated with poor prognosis. (**A**) UBQLN1 mRNA expression levels of colorectal cancer samples and normal control were analyzed by quantitative RT-PCR; (**B**,**C**) relapse-free survival (RFS) of colon cancer patients (**B**) and colorectal cancer patients (**C**) based on UBQLN1 mRNA expression was analyzed by lnCAR database. Data are shown as mean ± SD; *** *p* < 0.001 based on Student’s *t*-test.

To assess the overall profile of UBQLN1 expression in colorectal cancer, we analyzed the UBQLN1 gene expression level in a Gene Expression Omnibus (GEO) dataset (GSE106582), and these data were analyzed via a scatter plot. UBQLN1 showed significantly increased expression in 77 colorectal cancer tissues compared with 117 healthy control tissues (Figure 1A).

We used the lnCAR database (https://lncar.renlab.org/, accessed on 7 August 2020) to access prognosis data for those colorectal patients with high levels of UBQLN1 expression. Prognostic values, including two relapse-free survival (RFS) values of UBQLN1 mRNA expression, respectively, in colon cancer samples and colorectal cancer tissues, were estimated. We found that the mRNA expression level of UBQLN1 was negatively correlated with RFS in colon cancer (Figure 1B; analysis ID: CR_O19, *p* = 0.0056) and colorectal cancer (Figure 1C; analysis ID: CR_O16, *p* = 0.0172).

### 3.2. UBQLN1 Promoted CRC Cell Proliferation In Vitro

**Figure 2:**
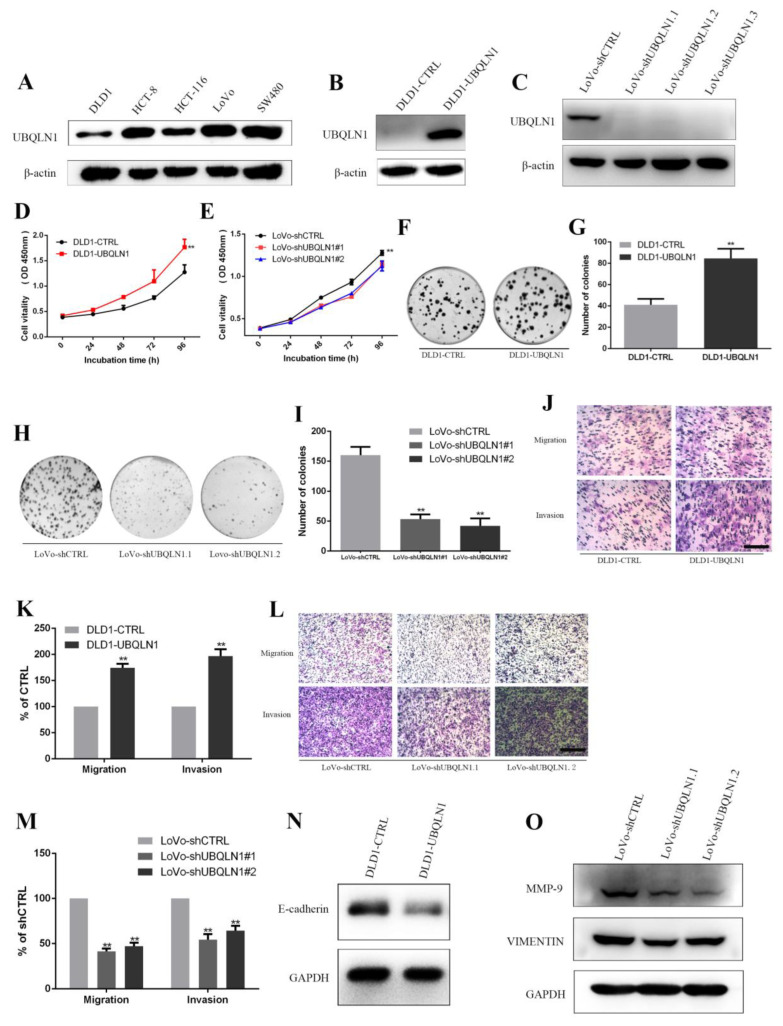
UBQLN1 promoted colorectal cancer cell progression in vitro. (**A**) UBQLN1 protein expression levels were detected in different cell lines by Western blot; (**B**) colorectal cancer cell line DLD1 was used to establish UBQLN1 over-expression cell line; (**C**) colorectal cancer cell line LoVo was used to establish UBQLN1 silencing cell line; (**D**,**E**) CCK-8 assay was performed to detect cell proliferation in DLD1-UBQLN1 (**D**) and LoVo-shUBQLN1 (**E**); (**F**,**G**) colony formation assay was performed in DLD1-UBQLN1 to detect cell colony formation ability and results were displayed as represent figures (**F**) and statistical graph (**G**); (**H**,**I**) colony formation assay was performed in LoVo-shUBQLN1 to detect cell colony formation ability and results were displayed as represent figures (**H**) and statistical graph (**I**); (**J**,**K**) Transwell and Matrigel assays were performed in DLD1-UBQLN1 to detect cell vitality and mobility and results were displayed as represent figures (**J**) and statistical graph (**K**), and the scale bars represent 50 μm; (**L**,**M**) Transwell and Matrigel assays were performed in LoVo-shUBQLN1 to detect cell vitality and mobility, results were displayed as figures (**L**) and statistical graph (**M**), and the scale bars represent 50 μm; (**N**,**O**) Western blot was performed to detect EMT marker proteins in UBQLN1 over-expression cell line DLD1 (**N**) and UBQLN1 silencing cell line LoVo (**O**). Data are shown as mean ± SD; n = 3 independent experiments; and ** *p* < 0.01 based on Student’s *t*-test.

UBQLN1 protein expression level was measured in five CRC cell lines (DLD1, HCT-8, HCT-116, LoVo, and SW480) by Western blot, respectively (Figure 2A). The results showed that among five CRC cell lines, the expression level of three cell lines (HCT-8, LoVo, and SW480) was higher compared to another two (DLD1 and HCT-116).

To identify the functional role of UBQLN1 in CRC cells, we established two CRC cell lines that stably enhanced/reduced UBQLN1, whose expression level was assayed by Western blot (Figure 2B,C). CCK-8 assay and colony-formation assay were performed to examine the effect on the cell’s abilities of proliferation and colony formation brought by UBQLN1. As shown, DLD1-UBQLN1 and LoVo-shUBQLN1 cells, which increased/decreased UBQLN1, exhibited higher/lower ability of proliferation than that of control cells, i.e., DLD1-CTRL and LoVo-CTRL (Figure 2D,E), as well as the ability of colony formation (Figure 2F–I). 

### 3.3. UBQLN1 Promoted CRC Cells’ EMT In Vitro

To ascertain whether over-expression/knockdown of UBQLN1 would influence cell epithelial–mesenchymal transition (EMT) capacities, a transwell migration and invasion assay were performed. Over-expression of UBQLN1 increased the numbers of migrated and invaded cells through the bottom of the transwell with or without matrigel (Figure 2J,K). In contrast, the knockdown of UBQLN1 decreased migrated and invaded cells’ numbers (Figure 2L,M). Meanwhile, Western blot assay was used to detect EMT marker E-cadherin, VIMENTIN, and MMP-9. The protein level of E-cadherin was reduced in DLD1-UBQLN1 cells compared with the control level (Figure 2N), and the protein level of VIMENTIN and MMP-9 was reduced in LoVo-shUBQLN1 cells compared with the control level (Figure 2O), suggesting that UBQLN1 may also enhance CRC cells’ EMT.

### 3.4. Reduced UBQLN1 Inhibited CRC Cells’ Tumorigenesis and Metastasis In Vivo

**Figure 3:**
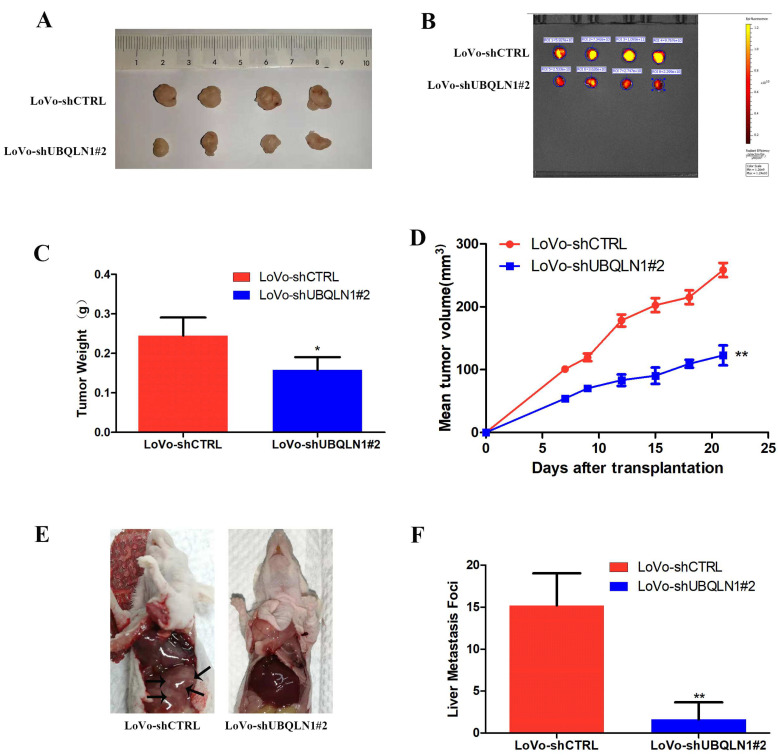
Reduced UBQLN1 inhibited CRC cells’ tumorigenesis and metastasis in vivo. *(***A**,**B**) Nude mice were subcutaneously injected with UBQLN1 silencing cell line LoVo and its control cells. The transplanted tumors were dissected out. (**C**) tumors’ weights were measured after dissection. (**D**) tumors’ volumes were measured during growth process. (**E**,**F**) nude mice were injected with UBQLN1 silencing cell line LoVo and its control cells through tail vein to establish distant metastasis model. The nude mice were sacrificed by anesthesia, and their liver metastasis was observed. Results were displayed as figures (**E**) and statistical graph (**F**). Data are shown as mean ± SD; n = 3 independent experiments; and * *p* < 0.0.5, ** *p* < 0.01 based on Student’s *t*-test.

To investigate the role of UBQLN1 in CRC carcinogenesis in vivo, a CRC xenograft model was established by implanting LoVo-shCTRL and LoVo-shUBQLN1 cells subcutaneously into the right flanks of nude mice. The results showed the tumors of the mice injected with LoVo-shCTRL cells after injection for four weeks (Figure 3A,B). The average weight of the tumors taken from the mice injected with LoVo-shCTRL cells was 245 milligrams, while that of the tumors from the ones injected with LoVo-shUBQLN1 cells was 158 milligrams (Figure 3C). Additionally, Tumor growth curves indicated that reduced UBQLN1 inhibited tumor growth in terms of volumes (Figure 3D).

Moreover, to detect the role of UBQLN1 in CRC metastasis in vivo, LoVo-shCTRL and LoVo-shUBQLN1 cells were injected into nude mice through the tail vein, respectively. The visible metastatic foci point in the liver of the mice injected with LoVo-shUBQLN1 cells was significantly less than those in the control group (Figure 3E,F). The results indicated that reduced UBQLN1 inhibited CRC cells’ tumorigenesis and metastasis in vivo.

### 3.5. Knockdown of UBQLN1 Suppressed ERK-c-Myc Signaling Pathway in CRC

**Figure 4:**
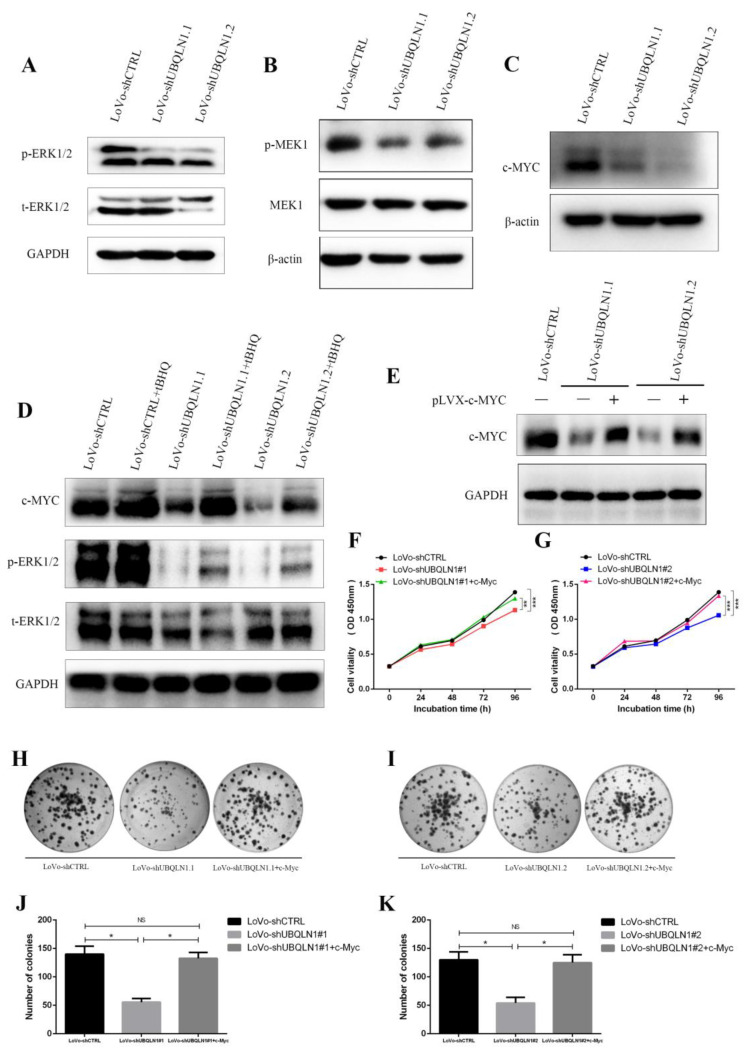
Knockdown of UBQLN1 inhibited colorectal cells’ malignant progression through ERK-c-Myc signaling pathway. (**A**,**B**) Western blot was performed to detect influence of UBQLN1 silencing on ERK-MAPK pathway; (**C**) Western blot was performed to detect influence of UBQLN1 silencing on expression of c-MYC; (**D**) Western blot was performed to detect relativity between ERK-MAPK pathway and c-MYC in UBQLN1 silencing cell line LoVo; (**E**) UBQLN1 silencing cell line LoVo was used to establish c-MYC over-expression cell line; (**F**,**G**) CCK-8 assay was performed to detect cell proliferation in LoVo-shUBQLN1 followed by c-MYC over-expression; (**H**–**K**) Colony formation assay was performed to detect cell colony formation ability in LoVo-shUBQLN1 followed by c-MYC over-expression; and results were displayed as figures (**H**,**I**) and statistical graph (**J**,**K**). Data are shown as mean ± SD; n = 3 independent experiments; and NS *p* ≥ 0.05, * *p* < 0.0.5, ** *p* < 0.01, and *** *p* < 0.001 based on Student’s *t*-test.

Approximately 50% of patients with metastatic colorectal cancer has mutations in the RAS gene, including HRAS, KRAS, and NRAS mutations, most of which are KRAS mutations [19,20,21]. KRAS mutations can abnormally activate the MAPK signaling pathway, causing the continuous activation of downstream ERK1/2 and promoting colorectal cancer malignant progression [22,23]. As we determined that the knockdown of UBQLN1 inhibited CRC cells’ progression both in vitro and in vivo, Western blot was used to detect the activity of ERK1/2, a downstream effector of RAS in the MAPK pathway. As shown, the phosphorylation level at site Thr202/Tyr204 of ERK1/2 protein was decreased, while the total protein level of ERK1/2 was changeless after knocking down UBQLN1 in CRC cells (Figure 4A). Meanwhile, Western blot was used to detect the activity of MEK1, an intermediator in signal transmission between RAS and ERK1/2. As shown, the phosphorylation level at site Thr286 of the MEK1 protein was decreased, while the total protein level of MEK1 was changeless after knocking down UBQLN1 in CRC cells (Figure 4B). Previous research has shown that c-Myc is a potential target of the ERK1/2-MAPK pathway [24]; thus, we detect the effect of UBQLN1 on c-Myc. We found that the protein level of c-Myc was decreased after knocking down UBQLN1 in LoVo cells (Figure 4C). Nextly, we sought to validate whether the knockdown of UBQLN1 reduces the expression of c-Myc through ERK-MAPK suppression. ERK1/2 activator tert-Butylhydroquinone (tBHQ, 50 μM) was used to treat LoVo-shUBQLN1 cells for 48 h. Western blot analysis showed activating the ERK signaling pathway reversed the decreased c-Myc protein expression brought by the knockdown of UBQLN1 (Figure 4D). Those results suggested the knockdown of UBQLN1 attenuated expression of c-Myc through the ERK1/2 signaling pathway.

### 3.6. Knockdown of UBQLN1 Inhibited CRC Cells’ Malignant Progression through ERK-c-Myc Signaling Pathway

To specify whether UBQLN1 loss inhibited CRC cells’ progression through the ERK-c-Myc signaling pathway, we overexpressed c-Myc in UBQLN1-deficient CRC cells (Figure 4E). The CCK-8 and colony formation assay showed LoVo cells’ abilities of proliferation and colony forming were restored to normal levels upon elevation of c-Myc in UBQLN1-deficient cells, establishing a strong connection between UBQLN1 and c-Myc (Figure 4F–K). We also confirmed the correlation between knockdown of UBQLN1 and ERK-c-Myc pathway in SW480 (Figure S1). These results confirmed that c-Myc is the downstream target of UBQLN1 that mediates proliferation by UBQLN1 loss. Above all, the knockdown of UBQLN1 inhibited CRC cells’ malignant progression through the ERK-c-Myc signaling pathway.

## 4. Discussion

UBQLN1 is a member of the UBQLN family, which plays important roles in protein degradation [7,25]. In our study, we found that over-expression of UBQLN1 promoted colorectal cancer cell progression, including proliferation, migration, and invasion, and vice versa. We also found knockdown of UBQLN1 downregulated the ERK-c-Myc pathway. Moreover, enhanced c-MYC rescued colorectal cancer cell progression caused by UBQLN1 silencing. To our knowledge, this is the first report to show that UBQLN1 played a role in colorectal cancer and was correlated with the ERK-c-Myc pathway.

As a member of the mitogen-activated protein kinases (MAPK) family, extracellular-signal-regulated kinases (ERK) are a type of serine/threonine protein kinase, including ERK1 and ERK2 [26]. The Ras/Raf/MEK/ERK signal transduction pathway follows the three-stage enzymatic cascade of MAPKs [27,28,29]. Ras acts as an upstream activating protein, which is activated after external stimulation and transmits the signal to Raf, namely MAPKKK. MEK acts as MAPKK to receive the signal [30]. Additionally, then the phosphorylated ERK is translocated to the nucleus, which mediates the transcriptional activation of Elk-1, c-fos, and c-Jun [29,31,32]. Finally, extracellular signals are transmitted to the nucleus, mediating cells to participate in a variety of life activities. Our study revealed that the knockdown of UBQLN1 inhibited the phosphorylation levels of ERK1/2 and MEK1, suggesting UBQLN1 could regulate the ERK pathway.

Previous studies have shown that activation of the ERK pathway could upregulate the expression level of c-Myc [24]. The *Myc* gene is the first proto-oncogene discovered in Burkitt lymphoma [33]. The *Myc* gene family members include *b-Myc*, *l-Myc*, *n-Myc*, *s-Myc,* and *c-Myc*, among which *c-Myc* is the most widely studied [34]. Previous studies observed the amplification or over-expression of the *c-Myc* gene in gastric cancer [35], breast cancer [36], cervical cancer [37], and other cancers, suggesting that the abnormal activation of the *c-Myc* gene is closely related to the occurrence and development of malignant tumors [38,39]. Similarly, our study revealed that the knockdown of UBQLN1 reduced protein expression of c-MYC and activated the ERK pathway, mediated by the tBHQ-rescued expression of c-Myc caused by UBQLN1 silencing.

Our study identified the knockdown of UBQLN1 down-regulated c-Myc by reducing the phosphorylation level of the ERK pathway. Furthermore, we found enhanced c-MYC rescued colorectal cancer cell progression caused by UBQLN1 silencing. These findings suggested that the knockdown of UBQLN1 inhibited colorectal cancer cell progression through ERK-c-Myc pathway.

## 5. Conclusions

The knockdown of UBQLN1 inhibits the progression of CRC through the ERK-c-Myc pathway, which provides new insights into the mechanism of CRC progression. UBQLN1 may be a potential prognostic biomarker and therapeutic target of CRC.

## Data Availability

Not applicable.

## Supplementary Materials

The following supporting information can be downloaded at https://www.mdpi.com/article/10.3390/cancers15123088/s1. Figure S1: Knockdown of UBQLN1 inhibited colorectal cells’ malignant progression through ERK-c-Myc signaling pathway; Figure S2: Densitometric analysis.
