# Supplementary material for: Knockdown of UBQLN1 Functions as a Strategy to Inhibit CRC Progression through the ERK-c-Myc Pathway
Source: Cancers (Basel). 2023 Jun 7;15(12):3088. doi: 10.3390/cancers15123088 (PMC10296216; doi:10.3390/cancers15123088)
Supplement: Supplementary file 1 [file cancers-15-03088-s001.zip › S1Knockdown of UBQLN1 inhibited colorectal cells malignant progression through ERK-c-Myc signaling pathway.pdf]

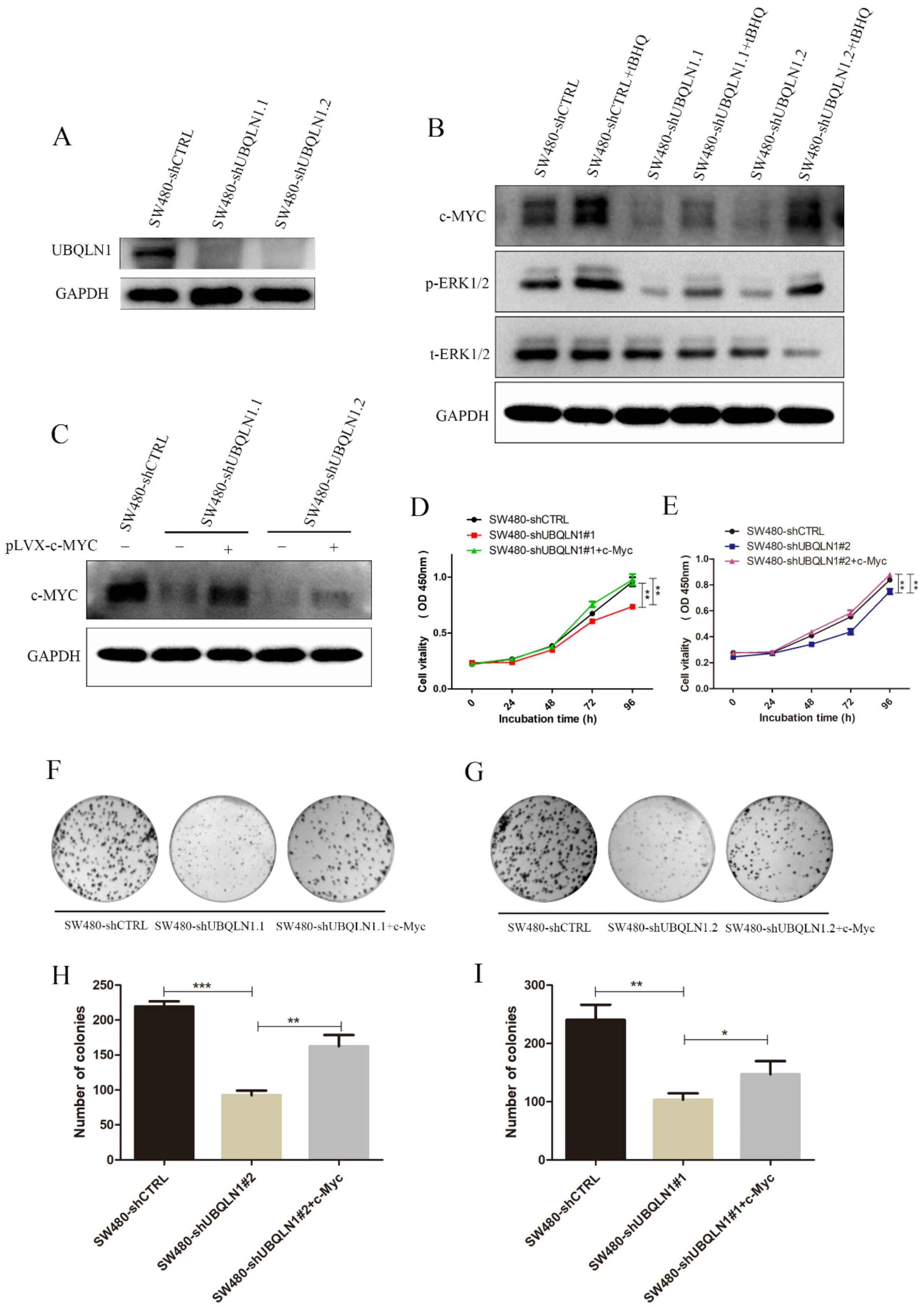

Fig.S1 Knockdown of UBQLN1 inhibited colorectal cells' malignant progression through ERK-c-Myc signaling pathway. **A** Colorectal cancer cell line SW480 was used to establish UBQLN1 silencing cell line; **B** Western blot was performed to detect relativity between ERK-MAPK pathway and c-MYC in UBQLN1 silencing cell line

SW480; **C** UBQLN1 silencing cell line SW480 was used to establish c-MYC over-expression cell line. **D,E** CCK-8 assay was performed to detect cell proliferation in SW480-shUBQLN1 followed by c-MYC over-expression; **F-I** Colony formation assay was performed to detect cells colony formation ability in SW480-shUBQLN1 followed by c-MYC over-expression and results were displayed as represent figures (**F,G**) and statistical graph (**H,I**). Data are shown as mean±SD; n=3 independndt experiments, * p < 0.0.5, ** p< 0.01, *** p< 0.001 based on the student's t test.
