# Supplementary material for: Knockdown of UBQLN1 Functions as a Strategy to Inhibit CRC Progression through the ERK-c-Myc Pathway
Source: Cancers (Basel). 2023 Jun 7;15(12):3088. doi: 10.3390/cancers15123088 (PMC10296216; doi:10.3390/cancers15123088)

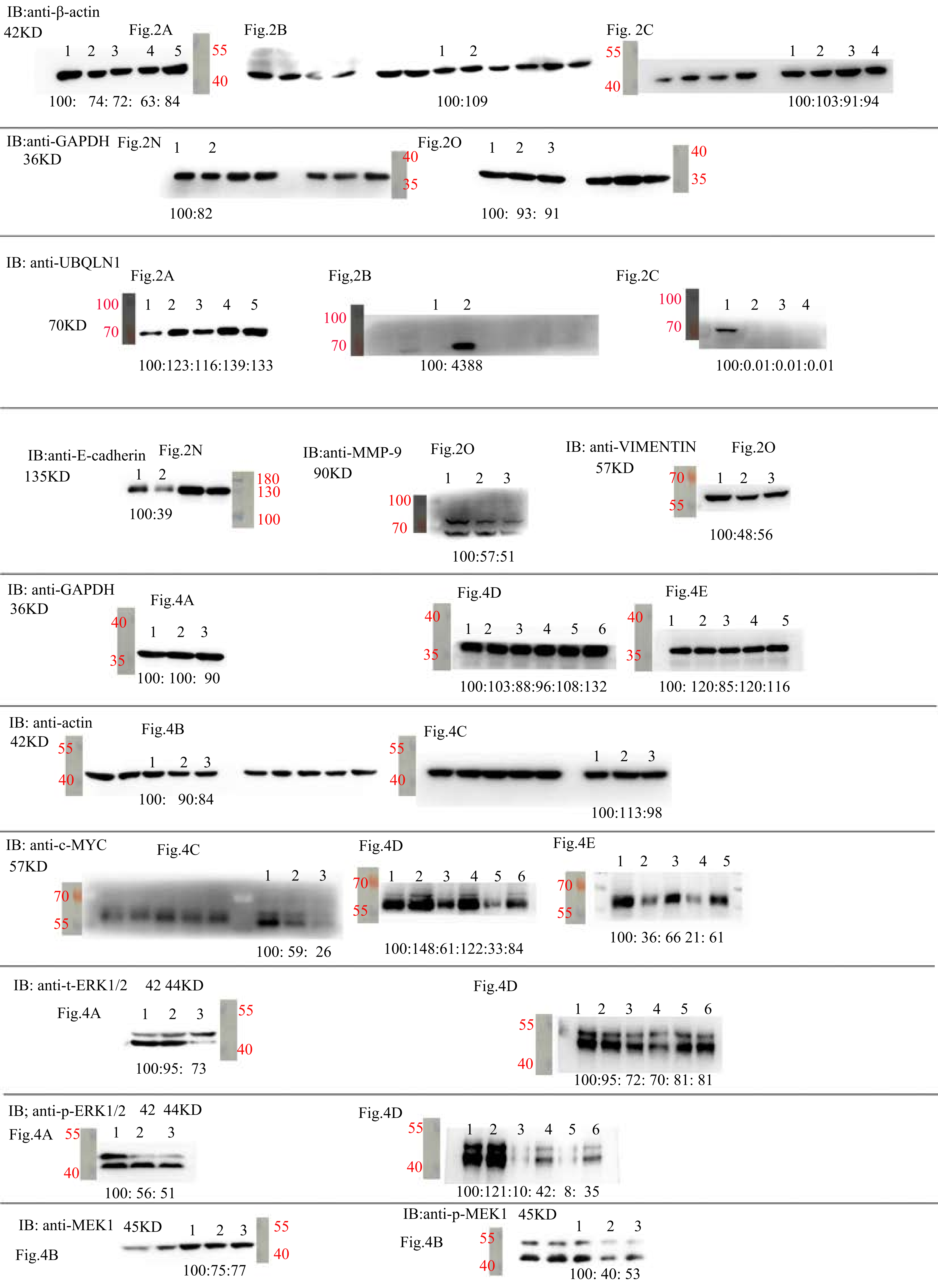
IB:anti-β-actin
42KD
Fig.2A
1 2 3 4 5
55
40
100: 74: 72: 63: 84
Fig.2B
1 2
100:109
Fig. 2C
55
40
1 2 3 4
100:103:91:94
IB:anti-GAPDH
36KD
Fig.2N
1 2
40
35
100:82
Fig.2O
1 2 3
40
35
100: 93: 91
IB: anti-UBQLN1
70KD
Fig.2A
100
70
1 2 3 4 5
100:123:116:139:133
Fig,2B
100
70
1 2
100: 4388
Fig.2C
100
70
1 2 3 4
100:0.01:0.01:0.01
IB:anti-E-cadherin
135KD
Fig.2N
1 2
180
130
100
100:39
IB:anti-MMP-9
90KD
Fig.2O
1 2 3
100
70
100:57:51
IB: anti-VIMENTIN
57KD
Fig.2O
70
55
1 2 3
100:48:56
IB: anti-GAPDH
36KD
Fig.4A
40
35
1 2 3
100: 100: 90
Fig.4D
40
35
1 2 3 4 5 6
100:103:88:96:108:132
Fig.4E
40
35
1 2 3 4 5
100: 120:85:120:116
IB: anti-actin
42KD
Fig.4B
55
40
1 2 3
100: 90:84
Fig.4C
55
40
1 2 3
100:113:98
IB: anti-c-MYC
57KD
Fig.4C
70
55
1 2 3
100: 59: 26
Fig.4D
70
55
1 2 3 4 5 6
100:148:61:122:33:84
Fig.4E
70
55
1 2 3 4 5
100: 36: 66 21: 61
IB: anti-t-ERK1/2 42 44KD
Fig.4A
1 2 3
55
40
100:95: 73
Fig.4D
55
40
1 2 3 4 5 6
100:95: 72: 70: 81: 81
IB; anti-p-ERK1/2 42 44KD
Fig.4A
55
40
1 2 3
100: 56: 51
Fig.4D
55
40
1 2 3 4 5 6
100:121:10: 42: 8: 35
IB: anti-MEK1 45KD
Fig.4B
1 2 3
55
40
100:75:77
IB:anti-p-MEK1 45KD
Fig.4B
55
40
1 2 3
100: 40: 53

IB:anti-GAPDH 36KD

Fig.S1A

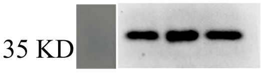

Fig.S1C

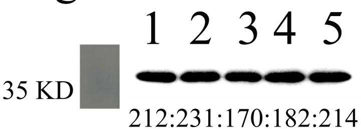

Fig.S1B

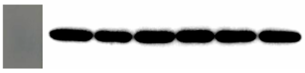

IB:anti-UBQLN1 70KD

Fig.S1A

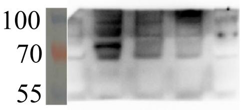

IB:anti-t-ERK1/2 42 44KD

Fig.S1B

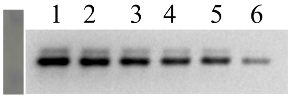

IB:anti-p-ERK1/2 42 44KD

Fig.S1B

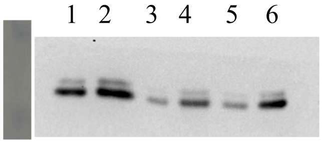

IB:anti-c-MYC 57KD

Fig.S1C

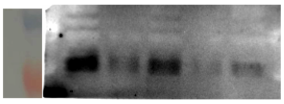

Fig.S1B

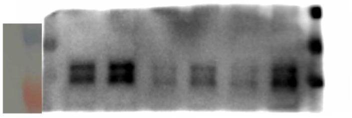

Supplement: Supplementary file 1 [file cancers-15-03088-s001.zip › S2 Densitometric analysis.pdf]
